# Time Trends of Gastrointestinal Cancers Incidence and Mortality in Yangzhong From 1991 to 2015: An Updated Age-Period-Cohort Analysis

**DOI:** 10.3389/fonc.2018.00638

**Published:** 2018-12-20

**Authors:** Yi Shao, Zhaolai Hua, Lei Zhao, Yi Shen, Xudong Guo, Chen Niu, Wenqiang Wei, Fen Liu

**Affiliations:** ^1^Department of Epidemiology and Health Statistics, School of Public Health, Beijing Municipal Key Laboratory of Clinical Epidemiology, Capital Medical University, Beijing, China; ^2^Department of Epidemiology, Yangzhong Cancer Research Institute, Yangzhong, China; ^3^Department of Molecular Physiology and Biophysics, Holden Comprehensive Cancer Center, University of Iowa Carver College of Medicine, Iowa City, IA, United States; ^4^National Cancer Center/Cancer Hospital, Chinese Academy of Medical Science and Peking Union Medical College, Beijing, China

**Keywords:** gastrointestinal cancers, incidence, mortality, age-period-cohort analysis, trends

## Abstract

**Background:** Gastrointestinal (GI) cancers are the common cause of morbidity and mortality in China which seriously threaten people's health and lives. The aim of this study was to describe the temporal trend in the epidemiology of GI cancers from 1991 to 2015, with an emphasis on the effects of age, period and cohort in Yangzhong City, Jiangsu province, a high-risk area of GI cancers in China.

**Methods:** Our study extracted cases of gastric cancer, esophageal cancer and colorectal cancer diagnosed from 1991 to 2015 from Yangzhong Cancer Registry. Age-standardized rates (ASRs) were calculated and joinpoint regression was used to compute the estimated annual percent changes. Age-period-cohort (APC) model was performed to investigate the independent effects of age, calendar period, and birth cohort.

**Results:** Between 1991 and 2015, 18,006 new cases and 10,262 deaths were registered with GI cancers in Yangzhong. The age-standardized incidence rates (ASIRs) of gastric cancer decreased in both sexes during the study period. And the incidence rates of esophageal cancer stabilized at first then continued to decline, the turning point was in 2005 for men and 2001 for women. Changes in the mortality rates of gastric cancer and esophageal cancer showed significant declined trends around 2000–2010 in both genders. The incidence rates of colorectal cancer increased steadily during the entire study period, and the increase was more pronounced in the mortality rates of men. The results of APC analysis suggest that general decreases in incidence and mortality of esophageal cancer and gastric cancer might be caused by the downward trend of the period and cohort effects, while the increases in colorectal cancer might be caused by the uptrend of the period effects.

**Conclusions:** The incidence and mortality rates of esophageal and gastric cancers showed a downward trend and colorectal cancer was on the rise as a whole in Yangzhong City. The different burden of gastrointestinal cancer indicating heterogeneous risk factors exist and may have contributed to these temporal variations.

## Introduction

Gastric cancer, esophageal cancer, and colorectal cancer are three most common types of gastrointestinal (GI) cancer, which is one of the most commonly diagnosed malignancies and one of the leading causes of cancer-related death worldwide, particularly in China (1, 2). It was estimated that 4,292,000 new cancer cases and 2,814,000 cancer-related deaths occurred in China in 2015, among which 35.72% (1,533,300 of 4,292,000) new cases and 37.81% (1,064,000 of 2,814,000) deaths were attributed to GI cancers (2). According to the National Central Cancer Registry of China (NCCRC) cancer statistics data in 2014, gastric cancer ranked the third highest incidence and the third most common cause of death, with estimated age-standardized incidence rate (ASIR) was 19.51/100,000 and an estimated age-standardized mortality rate (ASMR) was 13.3/100,000. Esophageal cancer is the sixth most common cancer and the fourth in cancer mortality, with ASIR and ASMR reaching as high as 12.17 and 8.75 per 1,00,000, respectively. Colorectal cancer had the fifth incidences and fifth mortality, the estimated ASIR and ASMR were 17.52 and 7.91 per 1,00,000, respectively (3).

In the year 2004, the Ministry of Health of China initiated a population-based endoscopy screening program in the high-risk areas of China (4). As a pilot rural area, massive population-based esophageal and gastric cancer screenings of asymptomatic patients have been performed in Yangzhong City (5). During the last several decades, with socioeconomic development and lifestyle changes simultaneously, declining incidence and mortality rates were observed in gastric cancer and esophageal cancer; however, colorectal cancer has increasing incidence and mortality in the Chinese population (6–8). The overall trends in GI cancer incidence and prevalence can only help us in understanding the changes in the epidemiology of GI cancer, but it won't be able to uncover the potential etiology clue. The longitudinal trends in the epidemiology of GI cancer can be influenced by a variety of risk or protecting factors, such as changes in dietary composition, living styles, and implementation of screening programs (9). The age-period-cohort (APC) analysis plays an important role in understanding time-varying elements in epidemiology. It usually identifies patterns in cancer incidence or mortality rates from population-based count (numerator) and population (denominator) data, which are often retrieved from cancer registry databases such as SEER in the form of a table showing the numbers of cancer cases or cancer deaths (counts) and corresponding person-years at risk (population) for particular age groups and calendar time periods. This analysis can help us separate the independent effects of age, period and cohort patterns, and further explores factors affecting the incidence and mortality of GI cancers from a macro perspective, such as social conditions, economic changes, environmental development (10, 11). By analyzing the overall trends of GI cancer epidemiology with the APC model, we can effectively identify potential risk factors and further make recommendations for prevention and control strategies of GI cancers.

Therefore, in order to identify the trends in GI cancers incidence and mortality rates and to reveal new information about the potential etiology clue of GI cancers, we chose a relatively fixed population from Yangzhong City, Jiangsu Province, which is a high-risk area of GI cancers in China (12, 13) to analyzed the secular trends in the incidence and mortality rates of gastric cancer, esophageal cancer, and colorectal cancer from 1991 to 2015 with the APC model.

## Materials and Methods

### Source of Data

Sex- and age-specific incidence and mortality data of GI cancers were obtained from Yangzhong Cancer Registry, 1991–2015. A detailed description of the cancer registration and reporting system in Yangzhong City can be found in a previous publication (14). Briefly, registered cancer cases were coded using the International Classification of Diseases, 9th Revision (ICD-9) before 2001, and using the 10th Revision (ICD-10) since 2002. Esophageal cancer (ICD-9:150; ICD-10: C15), gastric cancer (ICD-9:151; ICD-10: C16), and colorectal cancer (ICD-9:153-154; ICD-10: C18-21) were included in the APC analysis. The ICD revisions have no effect on the temporal trends for the incidence and mortality of GI cancer in our study. In this study, the demographic data (age composition by sex) from 1991 to 2015 were collected from the Yangzhong Statistics Department. Since our study did not involve interaction with human subjects or personal identifying information, ethical approval, and informed consent were not required.

### Quality Control

Quality of registration data was assessed based on the criteria of “Guideline for Chinese Cancer Registration (15)” and “Cancer Incidence in Five Continents Volume IX” by International Agency for Research on Cancer/International Association of Cancer Registries (IARC/IACR) (16). Briefly, the proportion of morphological verification (MV%), the percentage of cancer cases identified with death certification only (DCO%), mortality to incidence ratio (MI), and the percentage of the diagnosis of unknown basis (UB) (%) were used to evaluate the completeness, validity, reliability, and comparability of the data. Data included in the final analysis should meet the following criteria: MV% was not lower than 66%, DCO% was lower than 15%, M/I was between 0.6 and 0.8, and the percentage of the diagnosis of unknown basis (UB) (%) was < 5.0%. In the present study, the data have high quality and completeness as the overall MV%, DCO%, and MI ratio were 71.23, 2.98, and 0.65%, UB% was 0.32%, respectively.

### Statistical Analysis

Age-standardized incidence and mortality rates of GI cancers were calculated using Segi's World Standard population as the standard population (17).

To evaluate the trends in the epidemiology of GI cancers, we used the Joinpoint Regression Program (18). The log-linear model (based on Poisson distribution) of the joinpoint regression analysis program was employed to estimate trends in the age-standardized incidences and mortality rates of GI cancers, and the results were expressed as the estimated annual percent changes (EAPC) and their 95% confidence intervals (CIs) for each period. Significant joinpoints were identified by the Monte Carlo permutation test and *p* < 0.05 represents a statistically significant difference at the junction (19).

For the APC analysis, the cancer incidence and mortality data in this study were collected in five successive 5-year periods from 1991–1995 to 2011–2015, and ten 5-year age groups, ranging from 35–39 years to 80–84 years. The goal of APC analysis was to distinguish and statistically estimate the unique effects associated with age, calendar period, and a birth cohort from cross-sectional data respecting historical changes in the risk of morbidity and mortality. Age effects reflect the factors that associated with different age groups. Period effects reflect the factors that affect all age groups simultaneously, while cohort effects reflect the long-lasting effects of factors that influence all age groups simultaneously. Moreover, conventional APC models fall into the class of generalized linear models (GLM) that can take various alternative forms. When it refers to the estimation of cancer data which follow Poisson distributions, the model can be written as a log-linear regression model:

$$\begin{array}{l} ln\left(E\left(M_{ijk}\right)\right)=\ln\left(\frac{D_{ijk}}{P_{ijk}}\right)=\mu +\alpha_{\text{i}}\;+\beta_{\text{j}}\;+\gamma_{\text{k}}\;+\varepsilon_{\text{i}j\text{k}} \end{array}$$

where α_*i*_, β_*j*_, and γ_*k*_ represented age, period, and cohort effect, respectively. In the full 3-factor model, one inherent identification problem associated with the APC analysis by the exact linear dependency between age, period, and cohort: period = age + cohort (20). A number of methods have been proposed to solve this problem and we utilized the Intrinsic Estimator (IE) algorithm to address the identification problem and to provide parameter estimates because of its superior estimation ability, non-biased approach, validity, and asymptotic features. The IE method based on the estimable functions and the singular value decomposition of matrices, which does not need to have reference categories for the age, period, and cohort coefficients (21). The initial results are expressed as logarithmic coefficients, which can be seen as the natural logarithm of rate ratio (22, 23). To compare the rate ratio across ages, periods, and birth cohorts, we calculated the exponential value of the estimated coefficients (rate ratio = exp(coef.) = e^coef.^) (24). The goodness of fit for age-period-cohort sub-models were estimated using the Akaike information criterion (AIC). In this study, the calculation process was implemented in Stata version 14.0 (StataCorp, College Station, TX, USA).

## Results

### Incidence

**Figure 1:**
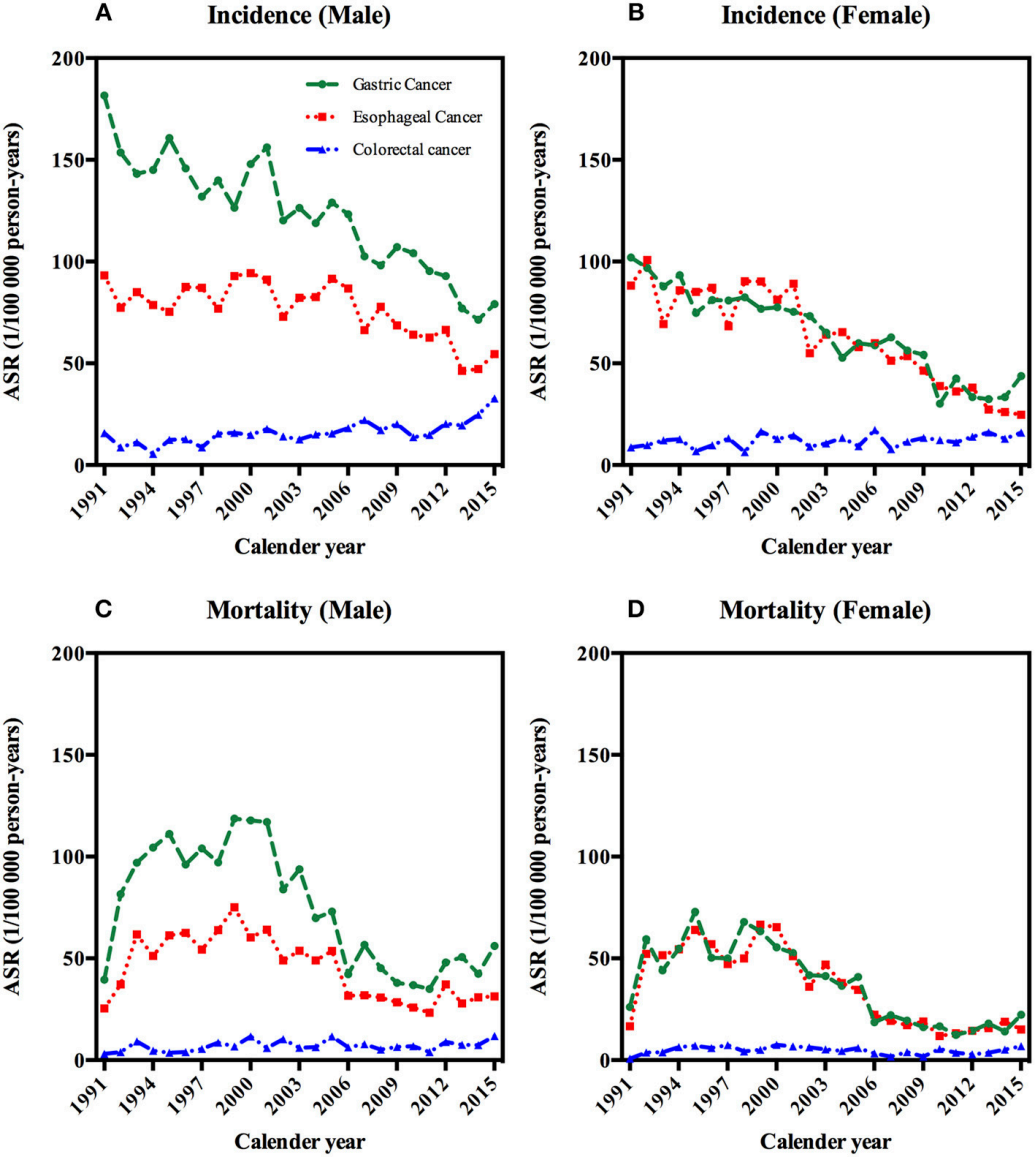
Age-standardized incidence and mortality rates of gastrointestinal cancers by sex in Yangzhong, 1991–2015. **(A)** Age-standardized incidence rates of gastrointestinal cancers in males. **(B)** Age-standardized incidence rates of gastrointestinal cancers in females. **(C)** Age-standardized mortaity rates of gastrointestinal cancers in males. **(D)** Age-standardized mortaity rates of gastrointestinal cancers in females.

A total of 9,400 gastric cancer cases, 7,107 esophageal cancer cases, and 1,499 colorectal cancer patients were registered during the period of 1991–2015 (Table 1). As shown in Figures 1A,B, ASIRs were highest in gastric cancer in men, followed by esophageal cancer and colorectal cancer in men. In women, the ASIRs of gastric cancer and esophageal cancer were almost at the same level, higher than that of colorectal cancer (Table 1; Figures 1A,B).

**Table 1:** Crude and age-standardized incidence and mortality rates (ASRs) of gastrointestinal cancers per 1,00,000 person-years in Yangzhong and calendar periods during 1991–2015.

**Calendar Periods**	**Incidence**						**Mortality**					
	**Males**			**Females**			**Males**			**Females**		
	***N***	**Crude rate**	**ASR**	***N***	**Crude rate**	**ASR**	***N***	**Crude rate**	**ASR**	***N***	**Crude rate**	**ASR**
**GASTRIC CANCER**												
1991–1995	1,301	189.28	156.76	797	114.88	90.90	702	102.13	86.79	469	67.60	51.47
1996–2000	1,133	164.85	138.39	706	101.82	79.74	856	124.55	106.71	538	77.59	57.35
2001–2005	1,238	180.24	128.81	682	97.65	64.60	818	119.09	86.18	460	65.86	42.18
2006–2010	1,210	177.28	106.76	647	92.47	51.93	496	72.67	43.65	244	34.87	18.59
2011–2015	1,122	162.33	82.81	564	78.71	37.05	650	94.04	46.56	274	38.24	16.27
**ESOPHAGEAL CANCER**												
1991–1995	696	101.26	81.89	746	107.53	85.84	399	58.05	47.41	430	61.98	47.79
1996–2000	741	107.82	87.76	735	106.00	83.40	515	74.93	63.23	526	75.86	57.18
2001–2005	809	117.78	83.90	700	100.23	65.09	509	74.11	53.42	467	66.87	40.82
2006–2010	820	120.14	72.42	617	88.18	49.36	331	48.50	29.54	239	34.16	17.75
2011–2015	752	108.80	55.05	491	68.52	30.27	422	61.05	30.19	275	38.38	15.55
**COLORECTAL CANCER**												
1991–1995	88	12.80	10.60	91	13.12	9.93	41	5.96	4.88	44	6.34	4.25
1996–2000	115	16.73	13.55	104	15.00	11.72	56	8.15	7.18	57	8.22	6.03
2001–2005	142	20.67	14.83	120	17.18	11.21	78	11.36	8.25	63	9.02	5.66
2006–2010	206	30.18	17.98	150	21.44	12.44	75	10.99	6.54	46	6.57	3.33
2011–2015	289	41.81	22.18	194	27.07	13.59	108	15.63	7.96	74	10.33	4.38
1991–1995	88	12.80	10.60	91	13.12	9.93	41	5.96	4.88	44	6.34	4.25

*ASR, age-standardized rates*.

Joinpoint regression analysis showed that ASIR of gastric cancer significantly decreased and without changes in the joinpoint regression by an average of 3.0% per year in men and 4.5% per year in women during 1991–2015. Regarding esophageal cancer, the ASIR in men has significantly decreased from 2005 to 2015 by an average of 5.4% per year; in women, the ASIR decreased even more rapidly by 7.7% per year from 2001 to 2015. On the contrary, the ASIR of colorectal cancer has increased steadily in both men (3.7%) and women (1.5%) from 1991 to 2015, again without change points during the entire observation period (Table 2).

**Table 2:** Joinpoint analysis of gastrointestinal cancers in Yangzhong, 1991–2015.

**Cancer types**	**Gender**	**Incidence**			**Mortality**		
		**Period**	**EAPC (95% CI)**	***P***	**Period**	**EAPC (95% CI)**	***P***
Gastric cancer	Male	1991–2015	−3.0 (−3.6, −2.5)	< 0.01	1991–1993	54.4 (3.1, 131.2)	< 0.01
					1993–2001	0.6 (−4.7, 6.2)	0.8
					2001–2010	−11.9 (−15.7, −8.0)	< 0.01
					2010–2015	9.5 (0.1,19.9)	< 0.01
	Female	1991–2015	−4.5 (−5.2, −3.7)	< 0.01	1991–1999	7.0 (−0.3,14.7)	0.1
					1999–2011	−13.0 (−16.7,−9.1)	< 0.01
					2011–2015	11.3 (−9.1,36.3)	0.3
Esophageal cancer	Male	1991–2005	0.2 (−1.1, 1.6)	0.7	1991–1993	49.0 (−1.9, 126.3)	0.1
					1993–2000	2.6 (−4.4, 10.1)	0.4
		2005–2015	−5.4 (−7.6, −3.3)	< 0.01	2000–2010	−9.1 (−12.5, −5.6)	< 0.01
					2010–2015	4.7 (−4.6,15.6)	0.3
	Female	1991–2001	−0.7 (−3.5, 2.1)	0.6	1991–1993	66.3 (5.3,162.7)	< 0.01
					1993–2000	0.9 (−6.6,9.0)	0.8
		2001–2015	−7.7 (−9.3, −6.1)	< 0.01	2000–2010	−13.9 (−17.4,−10.2)	< 0.01
					2010–2015	4.5 (−5.7,15.7)	0.4
Colorectal cancer	Male	1991–2015	3.7 (2.2, 5.2)	< 0.01	1991–2015	2.3 (0.3, 4.4)	< 0.01
	Female	1991–2015	1.5 (0.1, 3.0)	< 0.01	1991–1993	156.1 (−18.6, 705.9)	0.1
					1993–2015	−2.6 (−5.2, 0.1)	0.1

*EAPC, estimated annual percent changes; CI, confidence interval*.

**Figure 2:**
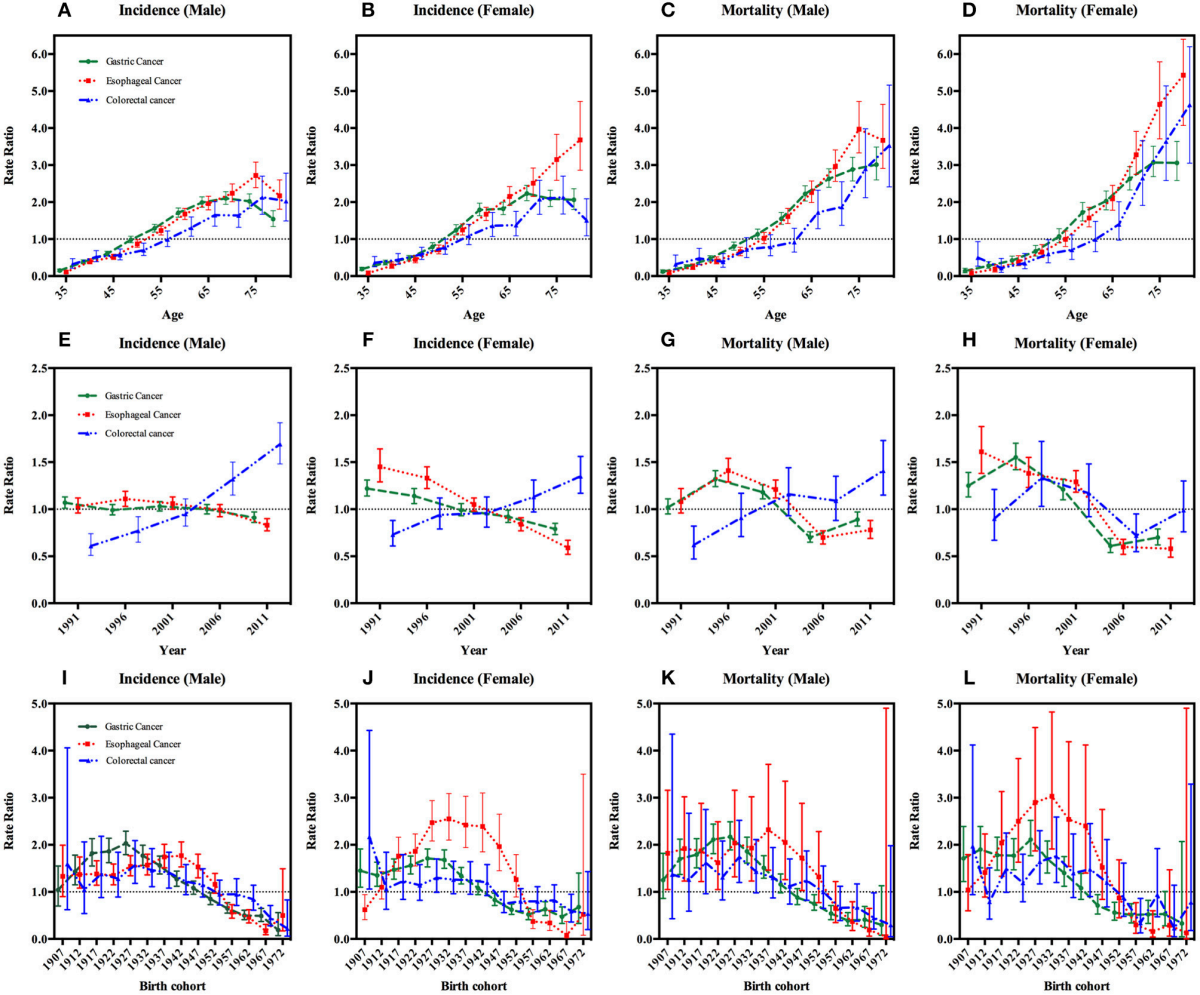
Age, period, and cohort effects on gastrointestinal cancers incidence and mortality with 95% confidence interval, stratified by sex. Footnotes: The age, period and cohort effects are estimated by a log-linear model using the intrinsic estimator (IE) method and expressed as rate ratios. **(A–D)** In the first row represent age effects after adjusting for period and cohort effects. **(E–H)** In the second row are the estimated period effects after adjusting for age and cohort effects. In the year 2004, a population-based endoscopy screening program in high-risk areas was initiated. **(I–L)** In the last row are the cohort effects after adjusting for age and period effects.

We then carried out the full APC models based on the goodness of the fit (Table 3). Supplementary Table 1 shows the results from the estimation of the full APC models using the IE method. The model-derived age, period and cohort effects were expressed in terms of rate ratio and shown in Figures 2A–B,E–F, I–J, respectively. After controlling the period and cohort effects, the age effects for both genders showed that the incidence rates of these three GI cancers had consistently increasing trends in incidence with age for people aged 35–74 year (Figures 2A–B).

**Table 3:** Akaike information criterion (AIC) of age-period-cohort sub-models for gastrointestinal cancers incidence and mortality, Yangzhong, 1991–2015.

**Sub-models**	**Gastric cancer**				**Esophageal cancer**				**Colorectal cancer**			
	**Incidence**		**Mortality**		**Incidence**		**Mortality**		**Incidence**		**Mortality**	
	**Male**	**Female**	**Male**	**Female**	**Male**	**Female**	**Male**	**Female**	**Male**	**Female**	**Male**	**Female**
Age-period-cohort	7.98	7.24	7.57	6.71	7.35	7.15	6.71	6.18	5.95	5.75	4.96	4.61
Age-drift	769.17	714.42	887.73	813.44	631.20	993.02	606.14	762.33	334.17	276.28	232.42	227.31
Age-period	489.28	430.97	453.00	368.98	522.95	609.57	402.82	431.81	287.29	275.44	230.83	223.79
Age-cohort	397.51	357.08	477.74	405.07	375.58	363.76	397.99	346.10	291.91	282.57	244.66	233.77

The period effects for the incidence of both gastric and esophageal cancer showed a steady decline and the decreased more rapidly in women than in men; while colorectal cancer showed an upward trend in both sexes during the period of 1991–2015 (Figures 2E–F).

With regard to birth cohort, we observed the effects of increased first and then decreased for GI cancers incidences after we adjusted the age and period effects. For gastric cancer and colorectal cancer, the cohort effects peaked around the year 1927 in both genders (Table 4 and Figures 2I,J). And the highest risk of esophageal cancer incidence was delayed by 5–10 years compared with gastric cancer and colorectal cancer, the apparent peak appearing around 1942 for male, but the highest value was observed in the 1932 cohort for female (Table 4 and Figures 2I,J).

**Table 4:** Highest cohort effects (Rate ratio) on GI cancers incidence and mortality rates and the corresponding birth cohorts.

	**Gastric cancer**		**Esophageal cancer**		**Colorectal cancer**	
	**Birth cohort**	**Rate ratio (95%CI)**	**Birth cohort**	**Rate ratio (95%CI)**	**Birth cohort**	**Rate ratio (95%CI)**
Incidence male	1927	2.03 (1.79, 2.29)	1942	1.77 (1.53, 2.06)	1927	1.57 (1.18, 2.09)
Incidence female	1927	1.71 (1.53, 1.91)	1932	2.55 (2.10, 3.09)	1927	1.30 (1.00, 1.70)
Mortality male	1927	2.17 (1.89, 2.49)	1937	2.32 (1.46, 3.71)	1927	1.74 (1.20, 2.52)
Mortality female	1927	2.11 (1.77, 2.52)	1932	3.03 (1.91, 4.82)	1932	1.76 (1.20, 2.59)

*CI, confidence interval*.

### Mortality

Between 1991 and 2015, 5,507 gastric cancer deaths, 4,113 esophageal cancer deaths, and 642 colorectal cancer deaths were reported in Yangzhong City (Table 1). The ASMRs of gastric cancer and esophageal cancer increased firstly and then declined, thereafter kept steady; while colorectal cancer remained stable in both sexes in 1991–2015 (Figures 1C,D and Table 1).

For gastric cancer, the ASMR in men increased by 54.4% on average per year in the period of 1991–1993, followed by a stable rate from 1993 to 2001, and then decreased by 11.9% on average per year from 2001 to 2010, and again an increase of 9.5% per year in the period of 2010–2015 was observed (Table 2 and Figures 1C,D). In women, the ASMR has been significantly decreased from 1999 to 2011 by 13% per year on average (Table 2 and Figures 1C,D). For esophageal cancer, the ASMR in men has been significantly decreased by 9.1% per year on average during 2000–2010. In women, the ASMR was increased during 1991–1993 (66.3% per year) and then reached a plateau from 1993 to 2000, followed by a decrease by 13.9% per year in the period of 2000–2010 (Table 2 and Figures 1C,D). Over the whole 25-year study period, there has been a significantly increasing trend of ASMR of colorectal cancer in male (2.3% per year), while female mortality trends will not present considerable variations (Table 2 and Figures 1C,D).

The full APC models of GI cancers mortalities had the lowest AIC value and were selected as the model with the best fit based on the goodness of the fit test results (Table 3). The age effects for both genders showed that the mortalities of gastric, esophageal and colorectal cancers had consistent increasing trends with age (Figures 2C,D).

In male, fluctuating period trends were presented in gastric cancer and esophageal cancer, which showed a rising trend first during the period of 1991–1995 to 1996–2000; then the period effects declined from the second 5-year period, i.e., from 1996–2000 to the period of 2006–2010; and then they increased thereafter up to the last period of 2011–2015. However, colorectal cancer as a whole showed an upward change in men. In the female, there were exhibited a fluctuating periodic pattern in the mortalities of gastric cancer and colorectal cancer, but a continuous decreasing period effect was observed in esophageal cancer (Figures 2G,H).

The cohort effect patterns of GI cancers in mortality were similar to those of incidence, showing an Inverted-U shape (Figures 2K,L). The cohort effect of gastric cancer peaked at around the year 1927 in both genders. The risk of colorectal cancer-related death in male was highest for those born in 1927, but in the female it was highest in 1932 cohort. And the risk of esophageal cancer-related death in male and female was highest for those born in 1937 and 1932, respectively (Table 4 and Figures 2K,L).

## Discussion

In this updated analysis of data from the 25-year period between 1991 and 2015, we observed divergent trends in the incidence and mortality of GI cancers in Yangzhong City. As a whole, the incidence and mortality rates of esophageal and gastric cancers showed downward trends while colorectal cancer was on the rise. Our APC analyses suggested that the incidence and mortality of GI cancers were increased exponentially with age. The fluctuated trends of GI cancers' incidence and mortality are primarily due to the changes of the period and cohort effects, which may indicate the impact of the early diagnosis and treatment of cancer, lifestyle and environmental changes on the risk of GI cancers in Yangzhong, a high-risk area of China.

### Age Effect

Similar age patterns were observed in the incidence and mortality trends of GI cancers in both sexes, from 35 to 74 years of age, indicating that the risk gradually increased with age. With modern medical technology and economic development, the proportion of elderly in the general population has increased dramatically; the burden caused by GI cancers would be a great challenge for China in the future. The average life expectancy was raised from 46 years in 1950 to 75 years in 2010 in China, and that of Jiangsu province is 1.6 years longer than the national level (76.6 years) (25). Elderly people are at a higher risk of developing GI cancers, which may be related to prolonged exposure to carcinogens. The rate ratio of GI cancers incidence and mortality was significantly increased from 40 years old and generally peaked in the age group of 75–79 years. In addition, women had the same level of the rate ratio of developing GI cancers compared to men. This indicated that cancer screening should be focused on those aged 40–74 years of both genders.

### Period Effect

The results of APC analyses showed that the trends of period effects in GI cancers were consistent with the age-standardized incidence and mortality rates trends, which indicated that the period effects might be an important factor affecting GI cancers morbidity and mortality. Variations in incidence and mortality over long periods often reflect the impacts of the changes in diet and lifestyle, updated diagnostic techniques, and improved medical interventions.

Since the late 1980s, the Yangzhong City government has paid attention to the prevention and treatment of cancers, especially esophageal cancer and gastric cancer. Under the leadership of the Institute of Cancer Research, a city-town-village tertiary cancer prevention and treatment network was established. And a comprehensive cancer prevention plan for education, environment, and medical care was established in 1998. Therefore, with the popularization of health education, some risk factors of upper GI cancers such as hot-temperature food, long-term stored rice, pickled vegetables, and drinking contaminated water have been greatly reduced (26, 27); meanwhile, the living and nutritional conditions of the local population also have been significantly improved. The incidence and mortality of esophageal and gastric cancer thereafter have changed significantly.

Furthermore, several screening methods have been developed and tested in the high-risk area of China. From 1986 to 1990, approximately 31,000 people in Yangzhong (age range, 35–70 years) received occult blood detection (28, 29). The latest study using endoscopic examination with Lugol's iodine staining and biopsy conducted in Yangzhong in the year 2004. With special funds from the Ministry of Health, local residents aged 40–69 years were eligible for a free endoscopic screening. Until 2012, 38,917 participants were covered from 26 villages in Yangzhong, which included 21,404 people age 40–69 years. In the target population, 13,888 participants were endoscopic examinations, and screening compliance was 64.89% (13,888 of 21,404 people). Among detected upper digestive tract cancers patients, 98.11% with esophageal cancer and 100% with gastric cancer were defined as at the early stage (ie, carcinoma *in situ* and intramucosal or submucosal [T1N0M0] carcinoma). Results from these studies showed that endoscopic screening and intervention significantly reduced mortality caused by esophageal or gastric cancer (5). This massive endoscopic screening program could largely explain the sharp downward trend of gastric and esophageal cancer mortality from the period of 2001–2005 to 2006–2010. Slight increases were observed in gastric and esophageal cancer mortality from 1991–1995 to 1996–2000, which might be mainly associated with the high prevalence of gastric cancer and esophageal cancer in the early 1990s and the underdevelopment of medical care during that period of time. In addition, a sudden tiny increase was captured in mortality of gastric and esophageal cancer, especially in male sex during the last period of 2011–2015. Reasons for this period effect fluctuation are not completely clear but may be related to aging, the high prevalence of H. pylori infection, and the frequent severe air pollution in China (30, 31). Besides, alcohol production and availability have increased over the past few years in China, and Chinese men have more social interactions to drink than women, which may also contribute to the burden of gastrointestinal cancer (32). There also might exit some potential risk factors which we still could not recognize and its harms outweigh the benefits of screening programs, further studies are needed to explore and primary prevention measures are still needed to reduce the exposure.

For colorectal cancer, the period effect was followed by a generally increasing trend in the incidence of both sexes and showed an upward trend in the mortality of the male. Previous studies based on extensive epidemiologic and experimental investigations have suggested that specific diet pattern, sedentary lifestyles, and obesity are associated with the increased risk of colorectal cancer (33, 34). With the accelerated economic development in Yangzhong, some westernized lifestyle such as excessive intake of high-protein and high-fat foods and less physical activity may contribute to the persistently rising incidence rate of colorectal cancer (35). In addition, some studies have shown higher zinc intake was significantly associated with reduced risk of colorectal cancer (36). According to a national representative cross-sectional study on nutrition and health in China in 2002, dietary zinc intake does not meet the Chinese Dietary Reference Intakes (DRIs) in Jiangsu Province (37), which might be one of the reasons for the rising incidence of colorectal cancer.

The gender difference in colorectal cancer mortality probably related to different exposures to risk factors in male and female. Some colorectal cancer risk factors such as smoking, diet, and obesity have been shown to have disparate effects on sex which may be related to interactions between estrogen exposure, body fat distribution, and the biologic underpinnings of the tumors (38). In addition, significant differences in colorectal cancer survival between men and women were demonstrated in some researches, indicating that higher survival rates persisted in women, which may partly explain the diversity in gender (39). Some studies have speculated that differences in immunological and genomic backgrounds could contribute to the gender-related differences in survival (40, 41). However, the underlying etiology clues driving gender differences regarding colorectal cancer mortality remains to be investigated.

### Cohort Effect

The birth cohort effect usually reflects the exposure of early life to specific risk factors that do not exist in other periods (42). Common risk factors such as tobacco smoking and diet may account for the long-term trend according to the similarity of the cohort patterns (43, 44). In our study, a noteworthy phenomenon is that the rate ratio for GI cancers incidence and mortality exhibited highest among those born in the late 1920s to the early 1940s. A possible explanation is that natural disasters have occurred during that period, followed by severe famine and deaths. At the beginning of the Twentieth century, social conditions and public health conditions in China were poor. Low socioeconomic status during childhood may partly explain the result. Individuals with poor socioeconomic status during childhood are characterized by a short length of life, high mortality rates, and independent of subsequent socioeconomic conditions (45, 46). A Study has suggested a possible link between exposure to the Chinese famine during childhood and an increased risk of GI cancers (47). So the newborns who survived those famines may have suffered from malnutrition and have a greater risk of developing digestive diseases. Esophageal cancer and gastric cancer have a relatively higher rate ratio than colorectal cancer during this period, meanwhile, the rate ratio of esophageal cancer was lower in men than in women. So, it can be inferred that esophageal cancer and gastric cancer have a stronger biological susceptibility to malnutrition, especially in women.

Since the early 1930s, the cohort effects on the incidence and mortality rates of gastric cancer and colorectal cancer has been declining, and esophageal cancer has declined since the early 1940s. Subsequently, the cohort effects of GI cancers all have dropped to low levels around the 1950s, which can be explained by improvements in socioeconomic status after World War II and the founding of new China (48). Furthermore, a relevant decrease in the prevalence of tobacco and alcohol consumption among the younger generation could be another possible reason. A study on the prevalence of smoking among different generations in China shows that the rate of smoking has decreased gradually from the birth population in the 1950s to the birth population in the 1980s (49). In addition, the decrease in the number of frequent alcoholics in rural China from those born in the 1940s may partially affect the decline in cohort effect (50).

### Strengths and Limitations

The present study has some strengths and limitations. Based on a high-quality population-based of cancer registration database, we conducted a comprehensive analysis of GI cancers incidence and mortality over a time period of 25 years in Yangzhong City, a high-risk area of GI cancers in China. Furthermore, APC analysis can effectively disentangle the separate effects on secular trends compared to traditional statistical methods. Our study also has several limitations. First, due to sample size limitations, the findings of this study may not be representative of other Chinese populations. Second, like other APC model analyses of cancer (51, 52), we analyzed the incidence and mortality of GI cancers without distinguishing histological types. However, the histological types of GI cancers in China are relatively simple. For example, more than 90% of esophageal cancers in China are mainly esophageal squamous cell carcinomas (53), which partly reduce the influence of histological type on this study. Last, the changes in the age, period and cohort effects cannot be specific to which pathogenic factors are caused. Therefore, the APC model of estimated parameters can only provide clues for etiology studies. The relevant hypotheses proposed in this study still need further confirmation in future studies.

## Conclusions

The incidence and mortality rates of esophageal and gastric cancers showed a downward trend and colorectal cancer was on the rise as a whole, indicating heterogeneous etiologies between upper gastrointestinal cancers and colorectal cancer. Significant changes in the period effects of esophageal and gastric cancer suggest that early detection and treatment programs are effective in reducing mortality, while the increasing trend of colorectal cancer probably attributed to the increased risk factors associated with westernized lifestyle. The similarity of cohort effects supports the influences of early childhood nutritional deficiencies on the development of GI cancers. All results from the APC analysis in this study still need further confirmation with more relevant studies.

## Author Contributions

YSha, WW, and FL contributed conception and design of the study. ZH collected and organized the database. YSha, YShe, XG, and CN performed the statistical analysis. YSha and LZ draft the manuscript. FL and WW Revised and made the decision to submit for publication. All authors contributed to manuscript revision, read and approved the submitted version.

### Conflict of Interest Statement

The authors declare that the research was conducted in the absence of any commercial or financial relationships that could be construed as a potential conflict of interest.
