# Supplementary material for: Time Trends of Gastrointestinal Cancers Incidence and Mortality in Yangzhong From 1991 to 2015: An Updated Age-Period-Cohort Analysis
Source: Front Oncol. 2018 Dec 20;8:638. doi: 10.3389/fonc.2018.00638 (PMC6306425; doi:10.3389/fonc.2018.00638)
Supplement: Supplementary file 1 [file Table_1.docx]

Supplementary Material

Time trends of gastrointestinal cancers incidence and mortality in Yangzhong from 1991 to 2015: an updated Age-period-cohort analysis

Yi Shao ^1#^, Zhaolai Hua^2#^, Lei Zhao^3^, Deqiang Zheng^1^, Yi Shen^1^, Xudong Guo^1^, Chen Niu^1^, Wenqiang Wei^4*^, Fen Liu^1*^

*** Correspondence:** Fen Liu: liufen05@ccmu.edu.cn; Wenqiang Wei: weiwq@cicams.ac.cn

# Supplementary Tables

**Supplementary Table 1(A)** APC model analysis of gastric cancer incidence and mortality in Yangzhong, 1991-2015.

|  | Incidence | | | | | | | | |  | Mortality | | | | | | | | |
| --- | --- | --- | --- | --- | --- | --- | --- | --- | --- | --- | --- | --- | --- | --- | --- | --- | --- | --- | --- |
|  | Male | | | |  | Female | | | |  | Male | | | |  | Female | | | |
|  | Coef | SE | 95% CI | |  | Coef | SE | 95% CI | |  | Coef | SE | 95% CI | |  | Coef | SE | 95% CI | |
| Age(year) | |  |  |  |  |  |  |  |  |  |  |  |  |  |  |  |  |  |  |
| 35-39 | -1.88 | 0.11 | -2.09 | -1.66 |  | -1.67 | 0.13 | -1.92 | -1.43 |  | -2.10 | 0.17 | -2.44 | -1.77 |  | -2.00 | 0.22 | -2.42 | -1.57 |
| 40-44 | -0.96 | 0.07 | -1.10 | -0.83 |  | -1.02 | 0.09 | -1.20 | -0.84 |  | -1.40 | 0.12 | -1.63 | -1.16 |  | -1.26 | 0.16 | -1.57 | -0.95 |
| 45-49 | -0.51 | 0.06 | -0.62 | -0.40 |  | -0.73 | 0.08 | -0.89 | -0.57 |  | -0.76 | 0.09 | -0.94 | -0.59 |  | -0.87 | 0.13 | -1.13 | -0.62 |
| 50-54 | -0.02 | 0.05 | -0.11 | 0.07 |  | -0.24 | 0.07 | -0.37 | -0.11 |  | -0.22 | 0.07 | -0.36 | -0.08 |  | -0.40 | 0.11 | -0.61 | -0.18 |
| 55-59 | 0.26 | 0.04 | 0.18 | 0.33 |  | 0.22 | 0.06 | 0.10 | 0.33 |  | 0.12 | 0.06 | 0.00 | 0.24 |  | 0.07 | 0.09 | -0.11 | 0.25 |
| 60-64 | 0.54 | 0.04 | 0.47 | 0.61 |  | 0.58 | 0.05 | 0.49 | 0.68 |  | 0.44 | 0.05 | 0.34 | 0.54 |  | 0.54 | 0.07 | 0.40 | 0.69 |
| 65-69 | 0.69 | 0.04 | 0.62 | 0.76 |  | 0.60 | 0.05 | 0.51 | 0.69 |  | 0.80 | 0.05 | 0.71 | 0.89 |  | 0.71 | 0.06 | 0.58 | 0.83 |
| 70-74 | 0.74 | 0.04 | 0.66 | 0.82 |  | 0.80 | 0.05 | 0.71 | 0.90 |  | 0.97 | 0.05 | 0.87 | 1.06 |  | 0.97 | 0.06 | 0.85 | 1.09 |
| 75-79 | 0.70 | 0.05 | 0.61 | 0.80 |  | 0.74 | 0.05 | 0.63 | 0.84 |  | 1.06 | 0.06 | 0.94 | 1.17 |  | 1.12 | 0.07 | 0.99 | 1.26 |
| 80-84 | 0.43 | 0.07 | 0.29 | 0.57 |  | 0.72 | 0.07 | 0.59 | 0.86 |  | 1.10 | 0.07 | 0.96 | 1.25 |  | 1.12 | 0.09 | 0.95 | 1.29 |
| Period(year) | |  |  |  |  |  |  |  |  |  |  |  |  |  |  |  |  |  |  |
| 1991-1995 | 0.07 | 0.03 | 0.01 | 0.13 |  | 0.20 | 0.04 | 0.13 | 0.27 |  | 0.02 | 0.04 | -0.06 | 0.10 |  | 0.23 | 0.05 | 0.12 | 0.33 |
| 1996-2000 | -0.01 | 0.03 | -0.06 | 0.05 |  | 0.13 | 0.04 | 0.06 | 0.20 |  | 0.28 | 0.03 | 0.21 | 0.35 |  | 0.44 | 0.04 | 0.35 | 0.53 |
| 2001-2005 | 0.03 | 0.03 | -0.02 | 0.08 |  | -0.01 | 0.04 | -0.08 | 0.06 |  | 0.17 | 0.03 | 0.10 | 0.23 |  | 0.19 | 0.04 | 0.10 | 0.28 |
| 2006-2010 | 0.00 | 0.03 | -0.05 | 0.05 |  | -0.08 | 0.04 | -0.15 | -0.01 |  | -0.36 | 0.04 | -0.44 | -0.28 |  | -0.49 | 0.06 | -0.61 | -0.38 |
| 2011-2015 | -0.09 | 0.03 | -0.15 | -0.03 |  | -0.24 | 0.04 | -0.32 | -0.16 |  | -0.12 | 0.04 | -0.20 | -0.03 |  | -0.36 | 0.06 | -0.48 | -0.24 |
| Cohort(year) | |  |  |  |  |  |  |  |  |  |  |  |  |  |  |  |  |  |  |
| 1907-1911 | 0.04 | 0.20 | -0.35 | 0.44 |  | 0.37 | 0.14 | 0.10 | 0.65 |  | 0.23 | 0.19 | -0.15 | 0.60 |  | 0.53 | 0.17 | 0.20 | 0.87 |
| 1912-1916 | 0.36 | 0.11 | 0.14 | 0.58 |  | 0.30 | 0.10 | 0.11 | 0.49 |  | 0.53 | 0.11 | 0.31 | 0.75 |  | 0.64 | 0.12 | 0.41 | 0.87 |
| 1917-1921 | 0.60 | 0.08 | 0.44 | 0.76 |  | 0.39 | 0.07 | 0.24 | 0.53 |  | 0.58 | 0.09 | 0.41 | 0.75 |  | 0.57 | 0.10 | 0.38 | 0.77 |
| 1922-1926 | 0.62 | 0.07 | 0.49 | 0.76 |  | 0.44 | 0.06 | 0.32 | 0.57 |  | 0.74 | 0.08 | 0.60 | 0.89 |  | 0.57 | 0.09 | 0.39 | 0.76 |
| 1927-1931 | 0.71 | 0.06 | 0.58 | 0.83 |  | 0.53 | 0.06 | 0.42 | 0.64 |  | 0.77 | 0.07 | 0.63 | 0.91 |  | 0.75 | 0.09 | 0.57 | 0.93 |
| 1932-1936 | 0.57 | 0.06 | 0.45 | 0.69 |  | 0.52 | 0.06 | 0.40 | 0.64 |  | 0.62 | 0.07 | 0.48 | 0.77 |  | 0.53 | 0.10 | 0.33 | 0.73 |
| 1937-1941 | 0.44 | 0.06 | 0.32 | 0.56 |  | 0.29 | 0.07 | 0.16 | 0.42 |  | 0.41 | 0.08 | 0.25 | 0.57 |  | 0.33 | 0.11 | 0.11 | 0.56 |
| 1942-1946 | 0.24 | 0.06 | 0.11 | 0.36 |  | 0.07 | 0.07 | -0.07 | 0.21 |  | 0.15 | 0.09 | -0.02 | 0.32 |  | 0.08 | 0.13 | -0.17 | 0.33 |
| 1947-1951 | 0.08 | 0.07 | -0.05 | 0.21 |  | -0.18 | 0.08 | -0.34 | -0.03 |  | -0.12 | 0.10 | -0.32 | 0.07 |  | -0.35 | 0.15 | -0.63 | -0.06 |
| 1952-1956 | -0.17 | 0.07 | -0.31 | -0.02 |  | -0.48 | 0.09 | -0.66 | -0.30 |  | -0.28 | 0.11 | -0.50 | -0.07 |  | -0.58 | 0.17 | -0.91 | -0.25 |
| 1957-1961 | -0.43 | 0.09 | -0.60 | -0.26 |  | -0.65 | 0.12 | -0.88 | -0.43 |  | -0.62 | 0.14 | -0.89 | -0.35 |  | -0.68 | 0.20 | -1.08 | -0.28 |
| 1962-1966 | -0.70 | 0.10 | -0.90 | -0.49 |  | -0.47 | 0.12 | -0.70 | -0.23 |  | -0.93 | 0.18 | -1.29 | -0.58 |  | -0.65 | 0.23 | -1.11 | -0.20 |
| 1967-1971 | -0.71 | 0.14 | -0.99 | -0.43 |  | -0.75 | 0.18 | -1.11 | -0.39 |  | -0.88 | 0.26 | -1.39 | -0.38 |  | -0.64 | 0.33 | -1.28 | 0.01 |
| 1972-1976 | -1.64 | 0.54 | -2.70 | -0.59 |  | -0.39 | 0.37 | -1.11 | 0.34 |  | -1.20 | 0.67 | -2.51 | 0.12 |  | -1.12 | 0.94 | -2.97 | 0.73 |

Coef: estimated coefficient; SE: standard error; CI: confidence interval.

**Supplementary Table 1(B)** APC model analysis of esophageal cancer incidence and mortality in Yangzhong, 1991-2015

|  | Incidence | | | | | | | | |  | Mortality | | | | | | | | |
| --- | --- | --- | --- | --- | --- | --- | --- | --- | --- | --- | --- | --- | --- | --- | --- | --- | --- | --- | --- |
|  | Male | | | |  | Female | | | |  | Male | | | |  | Female | | | |
|  | Coef | SE | 95% CI | |  | Coef | SE | 95% CI | |  | Coef | SE | 95% CI | |  | Coef | SE | 95% CI | |
| Age(year) | |  |  |  |  |  |  |  |  |  |  |  |  |  |  |  |  |  |  |
| 35-39 | -2.23 | 0.16 | -2.56 | -1.91 |  | -2.47 | 0.25 | -2.96 | -1.98 |  | -2.37 | 0.25 | -2.86 | -1.88 |  | -2.57 | 0.42 | -3.39 | -1.74 |
| 40-44 | -0.94 | 0.09 | -1.11 | -0.77 |  | -1.26 | 0.14 | -1.54 | -0.98 |  | -1.40 | 0.15 | -1.70 | -1.10 |  | -1.67 | 0.25 | -2.17 | -1.18 |
| 45-49 | -0.66 | 0.07 | -0.81 | -0.52 |  | -0.80 | 0.11 | -1.01 | -0.59 |  | -0.88 | 0.11 | -1.10 | -0.66 |  | -0.93 | 0.17 | -1.27 | -0.59 |
| 50-54 | -0.14 | 0.06 | -0.25 | -0.02 |  | -0.34 | 0.08 | -0.51 | -0.18 |  | -0.42 | 0.09 | -0.60 | -0.24 |  | -0.42 | 0.14 | -0.69 | -0.16 |
| 55-59 | 0.20 | 0.05 | 0.11 | 0.30 |  | 0.22 | 0.06 | 0.10 | 0.35 |  | 0.02 | 0.07 | -0.13 | 0.16 |  | -0.01 | 0.11 | -0.22 | 0.20 |
| 60-64 | 0.52 | 0.05 | 0.43 | 0.61 |  | 0.51 | 0.05 | 0.41 | 0.62 |  | 0.48 | 0.06 | 0.35 | 0.60 |  | 0.45 | 0.09 | 0.28 | 0.62 |
| 65-69 | 0.68 | 0.05 | 0.58 | 0.77 |  | 0.76 | 0.06 | 0.65 | 0.88 |  | 0.82 | 0.06 | 0.69 | 0.94 |  | 0.74 | 0.08 | 0.58 | 0.89 |
| 70-74 | 0.81 | 0.05 | 0.70 | 0.91 |  | 0.92 | 0.08 | 0.77 | 1.07 |  | 1.08 | 0.07 | 0.94 | 1.23 |  | 1.19 | 0.09 | 1.01 | 1.36 |
| 75-79 | 1.00 | 0.06 | 0.87 | 1.13 |  | 1.15 | 0.10 | 0.95 | 1.34 |  | 1.38 | 0.09 | 1.20 | 1.55 |  | 1.53 | 0.11 | 1.31 | 1.76 |
| 80-84 | 0.77 | 0.09 | 0.59 | 0.95 |  | 1.30 | 0.13 | 1.05 | 1.55 |  | 1.30 | 0.12 | 1.07 | 1.53 |  | 1.69 | 0.15 | 1.40 | 1.98 |
| Period(year) | |  |  |  |  |  |  |  |  |  |  |  |  |  |  |  |  |  |  |
| 1991-1995 | 0.03 | 0.04 | -0.04 | 0.11 |  | 0.37 | 0.06 | 0.25 | 0.49 |  | 0.08 | 0.06 | -0.04 | 0.20 |  | 0.48 | 0.08 | 0.32 | 0.63 |
| 1996-2000 | 0.10 | 0.04 | 0.03 | 0.17 |  | 0.29 | 0.04 | 0.20 | 0.37 |  | 0.34 | 0.05 | 0.25 | 0.43 |  | 0.32 | 0.06 | 0.21 | 0.44 |
| 2001-2005 | 0.06 | 0.03 | 0.00 | 0.13 |  | 0.05 | 0.03 | -0.02 | 0.12 |  | 0.19 | 0.04 | 0.11 | 0.27 |  | 0.26 | 0.05 | 0.17 | 0.35 |
| 2006-2010 | -0.01 | 0.03 | -0.08 | 0.05 |  | -0.18 | 0.04 | -0.27 | -0.09 |  | -0.36 | 0.05 | -0.47 | -0.26 |  | -0.52 | 0.07 | -0.65 | -0.38 |
| 2011-2015 | -0.18 | 0.04 | -0.26 | -0.11 |  | -0.53 | 0.07 | -0.66 | -0.40 |  | -0.25 | 0.06 | -0.37 | -0.13 |  | -0.54 | 0.09 | -0.71 | -0.37 |
| Cohort(year) | |  |  |  |  |  |  |  |  |  |  |  |  |  |  |  |  |  |  |
| 1907-1911 | 0.29 | 0.21 | -0.11 | 0.69 |  | -0.48 | 0.21 | -0.89 | -0.07 |  | 0.60 | 0.28 | 0.05 | 1.15 |  | 0.04 | 0.28 | -0.51 | 0.58 |
| 1912-1916 | 0.31 | 0.12 | 0.07 | 0.55 |  | 0.10 | 0.13 | -0.17 | 0.36 |  | 0.65 | 0.23 | 0.20 | 1.10 |  | 0.34 | 0.23 | -0.12 | 0.80 |
| 1917-1921 | 0.32 | 0.09 | 0.14 | 0.51 |  | 0.57 | 0.10 | 0.36 | 0.77 |  | 0.63 | 0.22 | 0.20 | 1.06 |  | 0.71 | 0.22 | 0.28 | 1.14 |
| 1922-1926 | 0.30 | 0.08 | 0.14 | 0.46 |  | 0.62 | 0.09 | 0.44 | 0.80 |  | 0.48 | 0.22 | 0.05 | 0.91 |  | 0.92 | 0.22 | 0.49 | 1.34 |
| 1927-1931 | 0.43 | 0.07 | 0.29 | 0.57 |  | 0.91 | 0.09 | 0.73 | 1.08 |  | 0.72 | 0.22 | 0.28 | 1.15 |  | 1.06 | 0.22 | 0.63 | 1.50 |
| 1932-1936 | 0.45 | 0.07 | 0.31 | 0.59 |  | 0.94 | 0.10 | 0.74 | 1.13 |  | 0.66 | 0.23 | 0.20 | 1.11 |  | 1.11 | 0.24 | 0.65 | 1.57 |
| 1937-1941 | 0.55 | 0.07 | 0.41 | 0.70 |  | 0.88 | 0.11 | 0.66 | 1.11 |  | 0.84 | 0.24 | 0.38 | 1.31 |  | 0.93 | 0.26 | 0.43 | 1.43 |
| 1942-1946 | 0.57 | 0.08 | 0.42 | 0.72 |  | 0.87 | 0.13 | 0.61 | 1.13 |  | 0.72 | 0.25 | 0.23 | 1.21 |  | 0.88 | 0.28 | 0.34 | 1.42 |
| 1947-1951 | 0.43 | 0.08 | 0.27 | 0.59 |  | 0.67 | 0.15 | 0.37 | 0.97 |  | 0.54 | 0.26 | 0.03 | 1.06 |  | 0.42 | 0.30 | -0.17 | 1.01 |
| 1952-1956 | 0.15 | 0.09 | -0.03 | 0.33 |  | 0.23 | 0.18 | -0.12 | 0.58 |  | 0.28 | 0.28 | -0.27 | 0.83 |  | -0.14 | 0.34 | -0.80 | 0.52 |
| 1957-1961 | -0.57 | 0.13 | -0.82 | -0.32 |  | -0.99 | 0.26 | -1.50 | -0.47 |  | -0.42 | 0.32 | -1.05 | 0.20 |  | -1.19 | 0.47 | -2.12 | -0.27 |
| 1962-1966 | -0.77 | 0.15 | -1.07 | -0.47 |  | -1.09 | 0.31 | -1.69 | -0.49 |  | -0.99 | 0.38 | -1.74 | -0.24 |  | -1.84 | 0.67 | -3.15 | -0.52 |
| 1967-1971 | -1.78 | 0.31 | -2.39 | -1.17 |  | -2.59 | 0.81 | -4.17 | -1.01 |  | -1.64 | 0.62 | -2.84 | -0.43 |  | -1.23 | 0.83 | -2.85 | 0.38 |
| 1972-1976 | -0.69 | 0.56 | -1.79 | 0.40 |  | -0.65 | 0.97 | -2.54 | 1.25 |  | -3.07 | 2.91 | -8.77 | 2.64 |  | -2.01 | 2.93 | -7.76 | 3.74 |

Coef: estimated coefficient; SE: standard error; CI: confidence interval.

**Supplementary Table 1(C)** APC model analysis of colorectal cancer incidence and mortality in Yangzhong, 1991-2015.

|  | Incidence | | | | | | | | |  | Mortality | | | | | | | | |
| --- | --- | --- | --- | --- | --- | --- | --- | --- | --- | --- | --- | --- | --- | --- | --- | --- | --- | --- | --- |
|  | Male | | | |  | Female | | | |  | Male | | | |  | Female | | | |
|  | Coef | SE | 95% CI | |  | Coef | SE | 95% CI | |  | Coef | SE | 95% CI | |  | Coef | SE | 95% CI | |
| Age(year) | |  |  |  |  |  |  |  |  |  |  |  |  |  |  |  |  |  |  |
| 35-39 | -1.15 | 0.20 | -1.54 | -0.76 |  | -1.01 | 0.19 | -1.39 | -0.64 |  | -1.15 | 0.30 | -1.74 | -0.56 |  | -0.69 | 0.31 | -1.30 | -0.08 |
| 40-44 | -0.65 | 0.15 | -0.94 | -0.37 |  | -0.82 | 0.17 | -1.14 | -0.49 |  | -0.73 | 0.23 | -1.18 | -0.28 |  | -1.51 | 0.40 | -2.30 | -0.73 |
| 45-49 | -0.56 | 0.13 | -0.82 | -0.31 |  | -0.52 | 0.14 | -0.80 | -0.25 |  | -0.95 | 0.23 | -1.41 | -0.49 |  | -1.05 | 0.29 | -1.62 | -0.49 |
| 50-54 | -0.35 | 0.12 | -0.59 | -0.12 |  | -0.27 | 0.14 | -0.53 | 0.00 |  | -0.33 | 0.19 | -0.70 | 0.04 |  | -0.52 | 0.25 | -1.02 | -0.02 |
| 55-59 | -0.01 | 0.11 | -0.22 | 0.20 |  | 0.09 | 0.13 | -0.16 | 0.34 |  | -0.24 | 0.18 | -0.59 | 0.11 |  | -0.35 | 0.23 | -0.80 | 0.10 |
| 60-64 | 0.27 | 0.10 | 0.07 | 0.47 |  | 0.31 | 0.12 | 0.07 | 0.54 |  | -0.09 | 0.17 | -0.43 | 0.25 |  | -0.01 | 0.20 | -0.41 | 0.39 |
| 65-69 | 0.50 | 0.10 | 0.30 | 0.70 |  | 0.32 | 0.12 | 0.09 | 0.56 |  | 0.54 | 0.15 | 0.25 | 0.84 |  | 0.33 | 0.19 | -0.03 | 0.70 |
| 70-74 | 0.50 | 0.11 | 0.28 | 0.72 |  | 0.73 | 0.11 | 0.51 | 0.95 |  | 0.63 | 0.16 | 0.32 | 0.94 |  | 0.97 | 0.17 | 0.65 | 1.30 |
| 75-79 | 0.75 | 0.12 | 0.51 | 0.99 |  | 0.76 | 0.12 | 0.52 | 0.99 |  | 1.07 | 0.16 | 0.75 | 1.38 |  | 1.29 | 0.18 | 0.95 | 1.64 |
| 80-84 | 0.71 | 0.16 | 0.40 | 1.02 |  | 0.41 | 0.17 | 0.09 | 0.74 |  | 1.26 | 0.19 | 0.88 | 1.64 |  | 1.53 | 0.21 | 1.12 | 1.95 |
| Period(year) | |  |  |  |  |  |  |  |  |  |  |  |  |  |  |  |  |  |  |
| 1991-1995 | -0.49 | 0.09 | -0.67 | -0.30 |  | -0.31 | 0.10 | -0.50 | -0.12 |  | -0.48 | 0.14 | -0.76 | -0.20 |  | -0.11 | 0.15 | -0.41 | 0.19 |
| 1996-2000 | -0.26 | 0.09 | -0.44 | -0.09 |  | -0.06 | 0.09 | -0.24 | 0.11 |  | -0.09 | 0.13 | -0.34 | 0.16 |  | 0.29 | 0.13 | 0.03 | 0.54 |
| 2001-2005 | -0.05 | 0.08 | -0.20 | 0.10 |  | -0.04 | 0.09 | -0.21 | 0.12 |  | 0.15 | 0.11 | -0.07 | 0.36 |  | 0.16 | 0.12 | -0.08 | 0.39 |
| 2006-2010 | 0.27 | 0.07 | 0.14 | 0.41 |  | 0.12 | 0.08 | -0.03 | 0.27 |  | 0.09 | 0.11 | -0.13 | 0.30 |  | -0.33 | 0.14 | -0.61 | -0.05 |
| 2011-2015 | 0.52 | 0.07 | 0.39 | 0.65 |  | 0.30 | 0.07 | 0.16 | 0.45 |  | 0.34 | 0.10 | 0.14 | 0.55 |  | -0.01 | 0.14 | -0.27 | 0.26 |
| Cohort(year) | |  |  |  |  |  |  |  |  |  |  |  |  |  |  |  |  |  |  |
| 1907-1911 | 0.46 | 0.48 | -0.48 | 1.40 |  | 0.78 | 0.36 | 0.06 | 1.49 |  | 0.31 | 0.59 | -0.85 | 1.47 |  | 0.68 | 0.38 | -0.06 | 1.42 |
| 1912-1916 | 0.05 | 0.34 | -0.62 | 0.72 |  | 0.07 | 0.27 | -0.47 | 0.61 |  | 0.23 | 0.38 | -0.52 | 0.98 |  | -0.23 | 0.33 | -0.88 | 0.42 |
| 1917-1921 | 0.32 | 0.23 | -0.13 | 0.78 |  | 0.20 | 0.19 | -0.18 | 0.58 |  | 0.49 | 0.27 | -0.04 | 1.01 |  | 0.40 | 0.21 | -0.01 | 0.81 |
| 1922-1926 | 0.25 | 0.19 | -0.12 | 0.61 |  | 0.13 | 0.17 | -0.20 | 0.46 |  | 0.27 | 0.24 | -0.19 | 0.73 |  | 0.17 | 0.21 | -0.24 | 0.59 |
| 1927-1931 | 0.45 | 0.15 | 0.17 | 0.74 |  | 0.26 | 0.14 | -0.01 | 0.53 |  | 0.55 | 0.19 | 0.19 | 0.92 |  | 0.49 | 0.17 | 0.15 | 0.83 |
| 1932-1936 | 0.38 | 0.14 | 0.10 | 0.65 |  | 0.23 | 0.14 | -0.04 | 0.50 |  | 0.36 | 0.20 | -0.02 | 0.75 |  | 0.57 | 0.20 | 0.18 | 0.95 |
| 1937-1941 | 0.35 | 0.14 | 0.08 | 0.61 |  | 0.22 | 0.14 | -0.05 | 0.49 |  | 0.27 | 0.20 | -0.13 | 0.67 |  | 0.34 | 0.24 | -0.13 | 0.80 |
| 1942-1946 | 0.20 | 0.13 | -0.06 | 0.46 |  | 0.18 | 0.14 | -0.09 | 0.45 |  | 0.11 | 0.21 | -0.30 | 0.53 |  | 0.39 | 0.26 | -0.11 | 0.90 |
| 1947-1951 | 0.14 | 0.13 | -0.11 | 0.40 |  | -0.29 | 0.15 | -0.58 | 0.01 |  | 0.21 | 0.21 | -0.20 | 0.63 |  | 0.18 | 0.29 | -0.39 | 0.75 |
| 1952-1956 | -0.05 | 0.14 | -0.32 | 0.22 |  | -0.23 | 0.15 | -0.52 | 0.07 |  | 0.00 | 0.22 | -0.43 | 0.44 |  | -0.13 | 0.31 | -0.73 | 0.48 |
| 1957-1961 | -0.05 | 0.15 | -0.34 | 0.25 |  | -0.22 | 0.17 | -0.54 | 0.11 |  | -0.41 | 0.27 | -0.94 | 0.11 |  | -1.08 | 0.48 | -2.02 | -0.15 |
| 1962-1966 | -0.18 | 0.16 | -0.49 | 0.13 |  | -0.19 | 0.17 | -0.53 | 0.14 |  | -0.40 | 0.28 | -0.96 | 0.16 |  | -0.06 | 0.36 | -0.76 | 0.65 |
| 1967-1971 | -0.81 | 0.24 | -1.28 | -0.34 |  | -0.54 | 0.25 | -1.02 | -0.05 |  | -0.79 | 0.39 | -1.56 | -0.02 |  | -1.48 | 0.82 | -3.09 | 0.13 |
| 1972-1976 | -1.51 | 0.68 | -2.84 | -0.19 |  | -0.62 | 0.50 | -1.59 | 0.36 |  | -1.21 | 0.96 | -3.10 | 0.68 |  | -0.25 | 0.74 | -1.70 | 1.19 |

Coef: estimated coefficient; SE: standard error; CI: confidence interval.
